# Point of Care Ultrasound (POCUS) in the Management of Heart Failure: A Narrative Review

**DOI:** 10.3390/jpm14070766

**Published:** 2024-07-18

**Authors:** Nicki Naddaf, Neda Dianati Maleki, Marc E. Goldschmidt, Andreas P. Kalogeropoulos

**Affiliations:** Division of Cardiology, Department of Medicine, Stony Brook Renaissance School of Medicine, Stony Brook, NY 11794, USA; nicki.naddaf@bmc.org (N.N.); neda.dianatimaleki@stonybrookmedicine.edu (N.D.M.); marc.goldschmidt@stonybrookmedicine.edu (M.E.G.)

**Keywords:** heart failure, point of care ultrasound, doppler echocardiography, B-lines, inferior vena cava, jugular vein, venous excess ultrasound

## Abstract

Assessing for volume overload is a key component of both short and long-term management of heart failure patients. Physical examination findings are neither sensitive nor specific for detecting congestion, and subclinical congestion may not be evident at the time of examination. Point of care ultrasound (POCUS) is an efficient and non-invasive way to assess heart failure patients for volume overload. The aim of our narrative review is to summarize how each of the following ultrasound modalities can be used to assess for congestion in the heart failure population: 2D and Doppler echocardiography, lung ultrasound, inferior vena cava ultrasound, internal jugular vein ultrasound, and venous excess grading. While each of these modalities has their limitations, their use in the acute and outpatient space offers the potential to reduce heart failure readmissions and mortality.

## 1. Introduction

As of 2023, approximately 6.7 million Americans have a diagnosis of heart failure [1]. A key player in heart failure pathology and prognosis is congestion, defined as pressure or volume overload in the circulatory system. Traditionally, clinical history and physical examination findings have been used to look for signs of underlying congestion in the heart failure population. However, these are neither sensitive nor specific measures of congestion. Furthermore, physical findings of congestion may manifest days to weeks after development [2,3]. Point of care ultrasound, also colloquially known as POCUS, is an efficient and non-invasive means to monitor for hemodynamic congestion in the heart failure population, especially in the setting of acute heart failure, and its use offers the potential to decrease rates of hospital admissions and all-cause mortality. The following provides an overview of various POCUS modalities and the existing literature documenting their use in the heart failure population, their limitations, and areas of possible future investigation.

## 2. Cardiac Ultrasound

Within the scope of cardiac ultrasound, transthoracic echocardiography (TTE), including two-dimensional (2D) and Doppler echocardiography, is a major tool in diagnosing and monitoring heart failure. In using TTE to calculate left ventricular ejection fraction (LVEF), new heart failure diagnoses are divided into heart failure with reduced ejection fraction (HFrEF, defined as LVEF ≤ 40%) or preserved ejection fraction (HFpEF, defined as LVEF ≥ 50%), with an intermediate category of mildly reduced ejection fraction (HFmrEF, LVEF between 40% and 50%) [4]. This is an important distinction as medical management differs according to LVEF in the chronic setting [3,5]. Of note, in the acute setting, these three entities share substantial similarities in presentation and management, as there are no therapies for acute heart failure specific to LVEF.

Among TTE, various protocols exist that vary in skill level, ranging from that of the novice ultrasound enthusiast to established echocardiographer. For example, the FoCUS exam is a popular protocol among emergency medicine clinicians that can be effectively performed with relatively little technical training [2]. The aim of the FoCUS exam is to provide answers to the “5 E’s,” which are defined as left ventricular EJECTION, presence or absence of cardiac EFFUSION, EQUALITY (size of the right ventricle), ENTRANCE (size and collapsibility of the inferior vena cava [IVC]), and EXIT (stroke volume calculated at the left ventricular outflow tract/cardiac output) [2,6].

**Figure 1:**
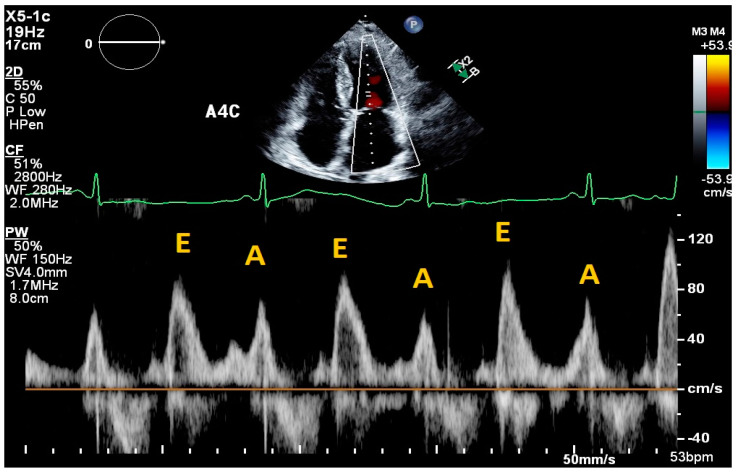
Mitral inflow velocities. Apical four chamber view. The pulse-wave Doppler sample volume is placed between the mitral valve leaflets at the level of the leaflet tips. Velocity tracing below. E wave corresponds to rapid ventricular filling. A wave corresponds to atrial systole.

While more technically challenging, cardiac ultrasound can also aid in the hemodynamic assessment of congestion by providing estimates for pulmonary capillary wedge pressure (PCWP), right atrial pressure (RAP), pulmonary pressures, and cardiac output (CO). Specifically, PCWP can be inferred based on echocardiographic measurements of left ventricular filling pressures. While there are a variety of methods to do this, one of the most used includes an assessment of diastolic transmitral inflow velocities using Doppler echocardiography (Figure 1). In this method, the pulse-wave Doppler sample volume is placed between the mitral valve leaflets at the level of the leaflet tips in the apical four chamber view to generate a velocity tracing. If obtained in a normal sinus rhythm, this tracing will include two waves: the E wave, which corresponds to rapid ventricular filling velocity, and the A wave, which corresponds to the peak filling velocity during atrial systole [7]. The E wave deceleration time (DT) is a measure that represents the time it takes for the maximum pressure gradient between the left atrium and ventricle to dissipate [2]. The existing literature has defined a normal DT to be between 160 and 200 ms, while a value of less than 140–150 ms is predictive of a PCWP greater than 15 mm Hg. A 2016 prospective cohort study which used the E wave DT and B-type natriuretic peptide (BNP) levels to guide diuretic therapy in outpatient HF patients noted a lower incidence of death (hazard ratio [HR] 0.45, 95% CI: 0.30–0.67, *p* < 0.0001) and lower incidence of worsening renal function (HR 0.49, 95% CI 0.36–0.67, *p* < 0.0001) compared to the control group (Table 1) [8]. However, there are several limitations in using E wave DT as a measure of congestion. DT is a poor estimate of left ventricular filling pressures in the HFpEF population [2,7]. Additionally, E and A waves may be absent or difficult to discern in cases of atrial fibrillation or flutter.

**Figure 2:**
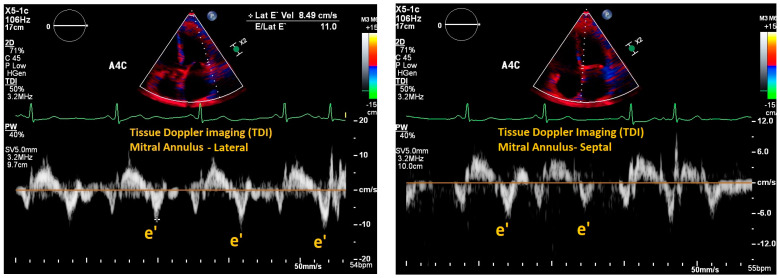
Lateral and septal mitral annular e’ velocities using tissue Doppler imaging (TDI). The e’ velocity corresponds to the velocity of tissue relaxation in early diastole. The average of lateral and septal mitral e’ velocities is used to calculate the E/e’ ratio which can be used to estimate LAP.

Another important parameter to estimate PCWP is the ratio of E velocity over the peak mitral annular velocity in the early diastole known as the e’ velocity. Annular velocities can be measured using tissue Doppler imaging (TDI) where the sample volume is placed over the mitral annulus and the velocity at which this structure moves during the cardiac cycle is recorded (Figure 2). This ratio is known as E/e’. The e’ measurement should be made from both the septal and lateral side of the annulus and the average should be used to calculate the E/e’ ratio [7]. E/e’ has been studied extensively to predict filling pressures and to estimate PCWP. The 2016 ASE/EACVI guidelines for the assessment of left ventricular diastolic dysfunction included E/e’ ratio as a diagnostic criteria for diastolic dysfunction and as a method to assess left ventricular filling pressures [7]. An E/e’ >14 is associated with elevated PCWP and left atrial pressures (LAP). Diagnosis of diastolic heart failure can be suggested on POCUS exam when the LVEF is estimated as >50% and left atrial pressure (LAP), as a surrogate for left ventricular end-diastolic pressure (LVEDP), is abnormally elevated.

The use of the E/e’ parameter may be limited in the presence of significant segmental wall motion abnormalities as well as the presence of prosthetic mitral valves, annular rings, and significant annular calcification [7]. Other parameters studied to estimate LV filling pressures include the ratio of mitral inflow velocities, left atrial volume index, and pulmonary venous flow; however, these methods are similarly technically challenging, have their own limitations of use, and lack a consensus for how they should be used among the heart failure population [2,3].

**Figure 3:**
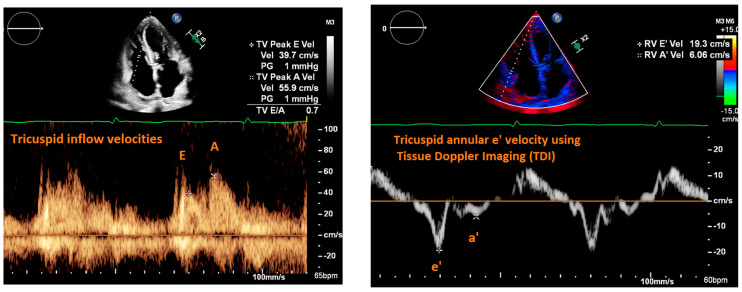
(**Left**): Tricuspid inflow velocities. Apical four chamber view. Pulse-wave Doppler sample volume is placed between the tricuspid valve leaflets at the level of the leaflet tips. Velocity tracing below. E wave corresponds to rapid right ventricular filling. A wave corresponds to the right atrial systole. (**Right**): Tricuspid annular velocity using tissue Doppler imaging. Tricuspid e’ corresponds to velocity of right ventricular tissue relaxation. Tricuspid E/e’ can be used to estimate RAP.

RAP can be measured using both 2D and Doppler echocardiography. The traditional way to estimate RAP using POCUS is by measuring IVC diameter and collapsibility (to be discussed in a later section). However, in cases where the IVC cannot be visualized, an alternative means for estimating RAP is through the examination of the tricuspid inflow velocity pattern [22]. Similar to the transmitral flow Doppler exam, the Doppler sample volume is placed between the tricuspid valve leaflets in the apical four chamber view to generate a tracing of the tricuspid valve inflow velocities in systole consisting of the E wave (rapid ventricular filling) and A wave (atrial systole) (Figure 3). Similar to the mitral valve, TDI can be used to measure the velocity of tissue relaxation of the lateral tricuspid annulus in diastole known as the tricuspid e’ velocity (Figure 3). The ratio of tricuspid E/e’ has been correlated to RAP; however, the level of correlation varies among different cardiac populations [9,22]. For example, Fletcher et al. conducted a systematic review which reported a consistently strong correlation between E/e’ and RAP in patients with right ventricular systolic dysfunction, but mixed support for using E/e’ to predict RAP in the heart failure population across the studies included (Table 1) [9]. Additional research is needed to determine the diagnostic utility to using the tricuspid inflow pattern, with and without correlating IVC measurements, to estimate RAP in the heart failure population as a whole. 

**Figure 4:**
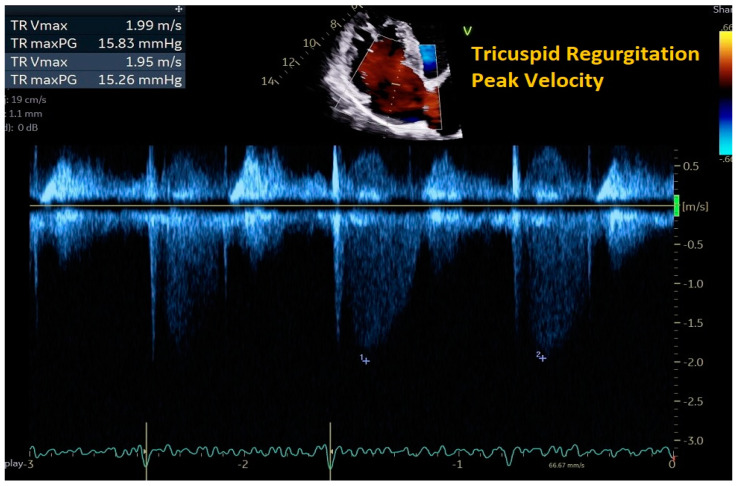
Estimation of systolic PA pressure (SPAP) with tricuspid regurgitation peak velocity. Using the modified Bernoulli equation, SPAP = (V^2^ × 4) + central venous pressure (CVP), where V is the tricuspid regurgitation peak velocity.

Estimates of pulmonary artery pressures (PAP) are useful in the treatment of HF as pulmonary hypertension is common in patients with HFrEF and HFpEF [23,24]. Although a component of the elevated pulmonary pressures may be attributed to pulmonary vascular remodeling, there is also a reversible component associated with the backward transmission of elevated left-sided pressures. Therefore, the absence of elevated PAP has a high negative predictive value for the absence of acute heart failure. Elevations in PAP have been shown to correlate with increased heart failure hospitalizations and higher rates of mortality, with decreases correlating with the opposite [23,25]. In decompensated HF, PAP tends to peak at the time of hospital admission and falls as patients undergo diuresis [26]. Doppler echocardiography can provide non-invasive estimates for systolic PA pressure (SPAP), diastolic PA pressure (DPAP), mean PA pressure (MPAP), and pulmonary vascular resistance (PVR) [22]. SPAP can be estimated using a modified version of the Bernoulli equation: (V^2^ × 4) + central venous pressure (CVP), where V is the tricuspid regurgitation (TR) maximal jet velocity (Figure 4). This method has been widely validated in patients with chronic heart failure. However, users must be wary of extremes in patient heart rate (bradycardia or tachycardia) and over- and underestimation of CVP, as these can lead to inaccuracies [22]. Another common limitation includes insufficient tricuspid regurgitation where TR jet velocity cannot be measured. DPAP and MPAP can similarly be estimated using the same formula, but with V in the equation derived from pulmonic regurgitation (PR) end diastolic jet velocity and PR maximal jet velocity, respectively. However, this method is not widely validated, and obtaining the PR jet on imaging is not always possible [22]. Additionally, a non-invasive estimate of pulmonary vascular resistance (PVR) can be approximated using the following formula: PVR (in Wood units) = [10 × maximal tricuspid regurgitation velocity/right ventricular outflow tract − velocity time integral (RVOT-VTI)] + 0.16. RVOT-VTI can be easily obtained by Doppler interrogation of the RVOT to measure the area under the curve of the velocity–time Doppler signal in systole. While this formula has been validated across several studies, it is not reliable in patients with PVR > 8 WU [22].

**Figure 5:**
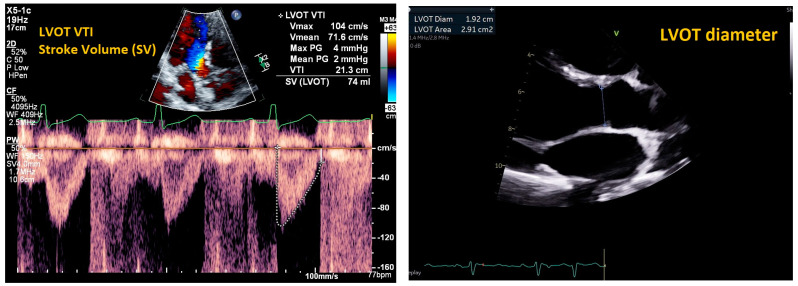
Measuring cardiac output (CO). CO = heart rate [HR] × stroke volume [SV]. Left panel: left ventricular outflow tract velocity time integral (LVOT VTI) in apical four chamber view. SV = *π* × R^2^ × (LVOT VTI). Right panel: diameter of the LVOT measured in parasternal long axis view in systole.

Cardiac output (CO) and cardiac index (CI) can also be non-invasively estimated using Doppler echocardiography (Figure 5). The definition of CO is heart rate (HR) × stroke volume (SV), and the CI is the CO divided by the patient’s body surface area [27]. By visualizing the parasternal long axis view, users can estimate SV by multiplying the cross-sectional area of the LVOT by the LVOT velocity time integral (VTI). This can also be represented as: SV = *π* × R^2^ × (LVOT VTI), where R is the radius of the LVOT mid-systole. In the absence of pulmonic shunting, users can also use the RVOT VTI and RVOT radius in mid systole to predict cardiac output [22]. By estimating cardiac output (CO) using LVOT Doppler interrogation, high cardiac output versus low cardiac output states can also be differentiated.

Although the gold standard for measuring CO/CI, pulmonary pressures, RAP, CVP, and PCWP remains right heart catheterization, Doppler echocardiography usually obviates the need for invasive testing for most patients with acute heart failure; the latter is usually reserved for critical presentations with severely depressed systolic blood pressure.

## 3. Lung Ultrasound (LUS)

Extravascular lung fluid is a hallmark of hemodynamic congestion, and lung ultrasound (LUS) offers an easy and efficient way to evaluate for the presence of pulmonary edema. In addition, LUS has been shown to be superior in detecting pulmonary edema compared to chest X-ray alone [2,28]. To perform LUS, users should select a low-frequency probe, point the probe marker cephalad, and aim the probe within the rib spaces [3]. There are several scanning zones in the literature, which range from a 2-zone hemi-thorax to the more traditional 28-zone scanning field [2].

Lung ultrasound relies on artifacts created by air–tissue interfaces. The most commonly described are A-lines and B-lines. A-lines are often described as a series of hyperechoic horizontal parallel lines, which are reverberation artifacts of the lung pleura [2]. The presence of A-lines suggests the absence of pulmonary edema, and also has been proposed to suggest a low pulmonary capillary wedge pressure (PCWP) [2,10]. Liechtenstein et al. demonstrated that an A-line-predominant lung pattern correlated with a PCWP of <13 mm Hg (90% specificity, 67% sensitivity, positive predictive value of 91%, and 65% negative predictive value) (Table 1) [10].

**Figure 6:**
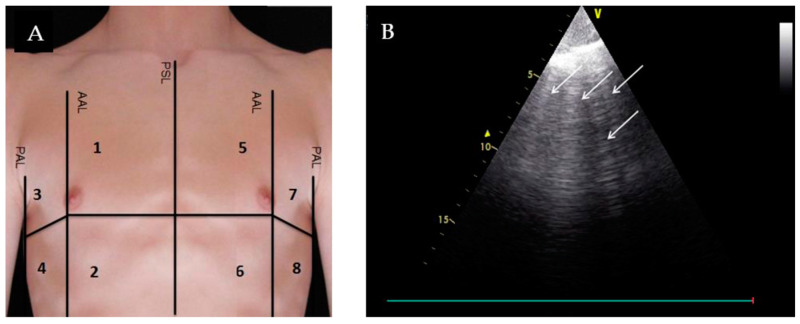
Detection of B-lines by lung ultrasound. (**A**) Division of the thorax in eight lung zones. PSL: parasternal line; AAL: anterior axillary line; PAL: posterior axillary line; (**B**) Example of a lung ultrasound loop of quadrant 1 at admission assessed as a positive region due to the appearance of ≥3 B-lines. Reproduced with permission from Glöckner et al., *Medicina* **2020**, 56, 379 [29].

In contrast, B-lines are described as comet-tail, hyperechoic artifacts that arise from the pleural line (Figure 6). The presence of multiple B-lines suggests pulmonary edema when correlated to the appropriate clinical context [2]. Imanishi et al. demonstrated increased B-lines count correlated with increased PCWP in patients with acute HFrEF and HFpEF and that acute HF patients with residual congestion (defined as the presence of >6 B-lines in an 8-zone scanning field) had a higher risk of subsequent cardiac events (hazard ratio 12.6; 95% confidence interval, 4.71–33.7; log-rank, *p* < 0.0001) (Table 1) [11].

While there is no consensus on the exact number, the presence of B-lines at discharge in acute heart failure patients has been shown to predict hospital readmission. Coiro et al. demonstrated that acute heart failure patients who had a B-line count ≥ 30 on 28-zone LUS at discharge were more likely to experience all-cause mortality or repeat HF hospitalization at 3 months (Table 1) [12]. Similarly, Dubón-Peralta et al. conducted a systematic review that studied the prognostic significance of B-lines on LUS at admission and discharge in patients hospitalized for acute HF (Table 1). They found that the presence of more than 30–40 B-lines at admission and persistent pulmonary congestion (defined as >15 B-lines) was similarly a strong predictor for all-cause mortality and hospital readmission [13].

In contrast to inpatient settings, there is not as strong of a consensus for the usefulness of LUS in managing chronic HF patients in the outpatient setting. Mhanna et al. conducted a systematic review and meta-analysis that showed that there was no significant difference in HF hospitalization rates and all-cause mortality in chronic HF patients who have LUS-guided management vs. standard of care alone (Table 1) [14]. In contrast, Li et al. conducted a meta-analysis that showed that LUS-guided management of chronic HF in outpatient settings correlated with a significantly lower risk of MACE compared to standard care (RR = 0.59; 95% CI = 0.48–0.71) (Table 1) [15]. Further research is needed on the effect of LUS-guided diuretic therapy in chronic HF patients.

## 4. Inferior Vena Cava (IVC) Ultrasound

**Figure 7:**
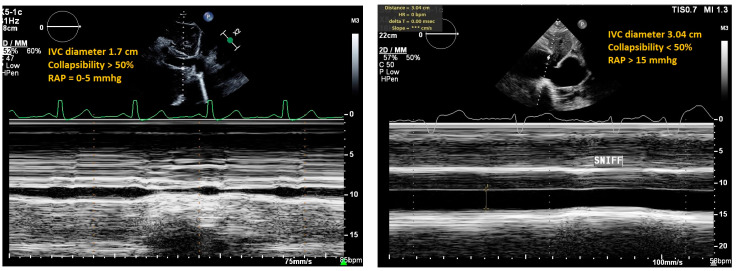
Normal (**left**) and dilated IVC (**right**). IVC diameter of less than 21 mm that collapses more than 50 percent with sniff suggests a normal right atrial pressure [RAP] (0–5 mm Hg). IVC diameter of greater than 21 mm with less than 50 percent collapsibility suggests elevated right atrial pressure (10–20 mm Hg). Left panel shows IVC diameter is 17 mm and collapses more than 50%, suggesting normal RAP. In contrast, the right panel has a measured IVC of 30.4 mm and collapsibility is >50%, suggesting elevated RAP.

Inferior vena cava (IVC) ultrasound is an efficient way to assess a patient’s volume status that requires low technical ability. To perform IVC ultrasound, users should choose a low-frequency probe, and with the probe marker cephalad, place the transducer in the subxiphoid region at a 90-degree angle [3]. Users can measure IVC diameter and can also ask the patient to “sniff” to evaluate IVC collapsibility. Because the right atrium is continuous with the IVC, IVC diameter and percent collapsibility can be used to predict right atrial pressure (RAP), and thus evaluate for venous congestion (Figure 7). In the literature, an IVC diameter of less than 21 mm that collapses more than 50 percent with sniff suggests a normal right atrial pressure (0–5 mm Hg); in contrast, an IVC diameter of greater than 21 mm with less than 50 percent collapsibility suggests elevated right atrial pressure (10–20 mm Hg) [30,31]. However, RAP cannot be estimated from IVC ultrasound measures in cases of increased intra-abdominal pressure, which notably include ileus, ascites, and mechanical ventilation [2]. 

While there are currently no guidelines for the use of IVC-guided diuresis in patients admitted with acute decompensated heart failure, there are several multi-center randomized controlled trials currently underway (Table 1). The CAVA-ADHF study aims to determine if the addition of IVC ultrasound to clinical assessment to guide diuretic therapy in hospitalized ADHF patients is superior to clinical assessment alone (NCT03140566). The JECICA study is investigating if daily bedside assessment of mitral inflow velocities and IVC measurements in ADHF patients would result in decreased 30-day readmissions and decreased mortality (NCT02892227). The CAVAL US-AHF aims to evaluate if IVC- and LUS-guided therapy in ADHF patients reduces subclinical congestion at discharge compared to standard of care (NCT04549701) [32].

In the outpatient setting, Curbello et al. measured the IVC collapsibility index (IVCCI), defined as the change in IVC diameter between inspiration and expiration divided by the maximum diameter (Table 1). In patients with an IVCCI of less than 30 percent, 70.9% experienced worsening HF compared to 39.1% of patients with an IVCCI of greater than 50 percent (HR 2.8 (95% CI: 1.3–6.2)). Additionally, 45.3% of patients with an IVCCI of less than 30 percent required admission compared to only five percent of patients with an IVCCI of greater than 50% (HR 13.9, 95% CI: 1.7–113.0) [16].

## 5. Internal Jugular Vein (IJV) Ultrasound

Clinical assessment of jugular venous pressure (JVP) is a hallmark clinical exam maneuver performed routinely in heart failure patients, as elevated JVP can indicate congestion. However, JVP evaluation can be limited by obesity, which is often comorbid with heart failure. Ultrasound of the internal jugular vein (IJV) is an easy way to evaluate for congestion that is not limited by body habitus.

To perform IJV ultrasound, users should select a high-frequency linear array probe and recline the patient at a 45-degree angle with the head and neck rotated to the side. If IJV visualization is difficult due to low CVP, patients can be asked to cough or perform the Valsalva maneuver to cause IJV engorgement. Once identified, there are several methods to measure the IJV. The JVD ratio involves taking the ratio of the maximum cross-sectional diameter of the IJV during Valsalva to the diameter at rest. The maximum diameter of the IJV during Valsalva is similar among patients with and without heart failure due to limited vessel compliance. However, IJV diameter at rest is generally small in patients with no heart failure diagnosis and increases in size as patients develop congestion. Thus, as at-rest IJV diameter increases, JVD ratio decreases [3]. The literature establishes a JVD ratio of less than 4 is abnormal and less than 2 may indicate severe congestion [17,33,34]. In outpatients, Pellicori et al. showed that low JVD ratio correlated with increased HF hospitalizations and incidence of death, independent of NT-proBNP measurements (Table 1) [17].

There are several studies that suggest IJV measurements can be used to assess for elevated CVP and RAP. Hossein-Nejad et al., conducted a prospective observational study in which they showed that the internal jugular vein/common carotid artery (IJV/CCA) ratio significantly correlated with CVP (r = 0.728, *p* < 0.0001 at inspiration, and r = 0.736, *p* < 0.0001 at expiration); for a CVP < 10, the IJV/CCA ratio had a sensitivity of 90%, specificity of 86.36%, positive predictive value of 90%, and negative predictive value of 86.36% (Table 1) [18]. Wang et al. similarly conducted a prospective observational study and showed that the upright assessment of jugular venous pressure with ultrasound was 94.6% specific for predicting elevated RAP (Table 1) [19]. Lastly, Vaidya et al., conducted a prospective cohort study that performed a supine assessment of IJV respiratory variation diameter (RVD) ([(D_max_ − D_min)_/D_max_] and collapsibility in heart failure patients prior to right heart catheterization (Table 1). Patients with an elevated RAP (>10 mm Hg) had less RVD (14% vs. 40% for IJV, *p* = 0.001) and reduced likelihood of total collapsibility with sniff (16% vs. 66%, *p* = 0.001). Lack of complete IJV collapsibility with sniff had an 84% sensitivity for elevated RAP [20].

## 6. Venous Doppler and Venous Excess Grading Ultrasound (VExUS)

An elevated right atrial pressure from a HF diagnosis translates to a congestion of abdominal organs and leads to poor health outcomes. Renal vein congestion is associated with progressive decrease in glomerular filtration rate, and hepatic venous congestion is associated with chronic congestive hepatopathy, which can predispose HF patients to ischemic hepatitis during a HF exacerbation [2]. To quantify systemic congestion, Beaubien-Souligny et al. developed a venous excess grading system (VExUS) using POCUS measurements of the IVC, hepatic vein Doppler, portal vein Doppler, and intra-renal venous Doppler (Table 1). The aim of their study was to use the VExUS prototype to predict acute kidney injury (AKI) in cardiac surgery patients and compare its clinical usefulness to CVP measurements. Severe venous congestion grade—defined as an IVC > 2 cm and the presence of severe flow abnormalities in multiple Doppler patterns—was significantly associated with the development of AKI (HR: 3.69 CI 1.65–8.24 *p* = 0.001), and it remained significant after adjustment for baseline AKI risk factors and vasopressor/inotropic medication use (HR: 2.82 CI 1.21–6.55 *p* = 0.02) [21].

## 7. Discussion

The number one cause for heart failure readmission is inadequate diuresis prior to discharge [35]. Elevated intracardiac and pulmonary artery pressures are associated with increased mortality in all types of cardiovascular disease with even mild elevations correlating to increased risk [11]. To date, there are no uniform discharge guidelines for heart failure patients other than physicians relying on physical exam findings and biomarkers. As discussed, physical exam findings often lack sensitivity and specificity to allow for precise determination of volume status [2,3]. Natriuretic peptides have improved the diagnosis and risk stratification of heart failure patients, but levels may be difficult to interpret based on confounding factors [3]. In addition, trials such as the Guiding Evidence-based Therapy Using Biomarker Intensified Treatment (GUIDE-IT) have not demonstrated improved outcomes by adjusting therapy based on serial measurements of natriuretic peptides [36,37].

In this paper we have explored the existing literature on the use of POCUS to non-invasively assess for hemodynamic congestion and guide treatment in the heart failure population. We summarize the common findings that suggest elevated left ventricular filling pressures across the various POCUS modalities in Table 2. An ultrasound protocol using 2D and Doppler echocardiography offers valuable hemodynamic information to determine volume status and guide treatment for heart failure patients. Specifically, we can non-invasively estimate cardiac output, PCWP, pulmonary artery pressures, and RAP (CVP) and thus obtain a complete hemodynamic assessment, often comparable with that of invasive monitoring. A recent meta-analysis of implantable hemodynamic monitoring systems that measured pulmonary artery pressures and left atrial pressures demonstrated a reduction in heart failure hospitalizations (HR 0.64, 95% CI: 0.55–0.76) and mortality (HR 0.75, 95% CI:0.57–0.99) over a median follow up of 12.2 months [23]. Given that POCUS can non-invasively make estimates of these measures, its systematic use in the heart failure population offers great potential to reduce hospitalizations and mortality.

A POCUS study performed by experienced clinicians in the emergency department in real time can assist in more efficient triaging of patients with dyspnea and edema. When decompensated heart failure is suspected, POCUS guides the clinical decision making and has the potential to decrease the time to disposition and to initiation of treatment, including intravenous diuretics. However, the data are not entirely concordant to date. In a retrospective cohort study among patients presenting to the emergency department with suspected heart failure and elevated B-type natriuretic peptide levels [38], the time to triage was substantially reduced (by a median of 2 h), as was the total length of hospital stay (by one-third), but the time to treatment increased by a median of 1.5 h. The latter has been partially attributed to the learning curve of implementing POCUS. In another emergency department study, however, lung POCUS led to faster treatment times for acute heart failure and chronic obstructive pulmonary disease (COPD) exacerbations [39]. Therefore, future studies are needed to systematically evaluate the role of the implementation of POCUS in reducing triage and treatment times and length of heart failure hospitalization.

Specifically for the assessment and therapy of acute heart failure, prospective studies have demonstrated that POCUS findings correlate with clinical assessments that incorporates levels of natriuretic peptides and that POCUS-based findings are strong prognostic indicators. In a study of 349 patients who received early LUS in the setting of acute heart failure [39], there was a strong correlation between the number of B-lines and natriuretic peptide levels. In the same study, the number of B-lines decreased with therapy, suggesting that LUS has the potential to track treatment response, and was independently associated with clinical events for up to 180 days. Importantly, over 95% of patients had interpretable images on admission and pre-discharge [40]. The prognostic value of LUS in acute heart failure has been also reported in a pooled analysis of international cohorts [41]. Combining POCUS modalities has the potential to further increase the efficacy of POCUS-based interventions. In a nurse-led study, the combination of lung and inferior vena cava ultrasound resulted in accurate assessment of pre-discharge residual congestion, as indicated by the strong predictive value of the ultrasound findings for 30- and 90-day readmissions [42]. However, prospective comparative studies are required to evaluate the effectiveness of combined POCUS interventions.

Currently, a wide range of commercially available POCUS ultrasound devices and transducers are available, ranging from compact portable consoles to handheld devices compatible with smartphones and electronic tablets. While all devices include basic two-dimensional and Doppler ultrasound features, differences in image quality depending on device and transducer characteristics may be present. Careful selection of ultrasound POCUS devices, based on the clinical practice setting, ease of use, portability, and cost, is critical.

Although POCUS has great potential to broadly differentiate disease entities in the acute setting, there should be a low threshold in obtaining a complete transthoracic echocardiogram whenever there is diagnostic ambiguity, or the diagnosis of heart failure is de novo. When the initial real-time POCUS exam is inconclusive or points towards non-cardiac etiologies of shortness of breath or edema, a full standard protocol transthoracic echocardiogram would be a more appropriate approach, complemented by other clinically indicated tests. In all, our group believes that POCUS is better suited to detect decompensation of known heart failure, guide acute treatment, and exclude obvious cardiac etiologies of presentation.

There are several challenges in the implementation of limited ultrasound for heart failure management, especially in the acute setting. First, although the implementation of limited ultrasound examination to guide acute care from providers of various specialties provides an opportunity for improved patient management, it also creates a set of issues associated with the standardization of clinical workflow and quality of care, as the implementation relies on local expertise and method availability. Second, as with most limited ultrasound techniques, protocolization is more challenging, as there is a substantial subjective element, which creates challenges related to wide implementation for clinical trial purposes. Third, the optimal approach among limited ultrasound techniques has yet to be determined, partially because of the challenges outlined above.

Ongoing clinical trials will elucidate the impact of limited ultrasound techniques on patient outcomes and provide evidence for the optimal approach, especially in acute settings. We recognize that there are certain challenges in the standardization of the acute process of care, as each healthcare system has a different workflow, which in turn could impact implementation of clinical trial protocols. On the other hand, we firmly believe that the only way forward is prospective studies with well-designed protocols, that would establish one or more POCUS modalities as effective interventions for the management of heart failure. The underlying rationale for well-designed trials is that methods that require training, time, and effort on behalf of the healthcare personnel, will only be integrated uniformly in practice when there is evidence of efficacy and cost-effectiveness.

## Figures and Tables

**Table 1:** Summary of select studies evaluating the use of point of care ultrasound for the management of heart failure.

Study	Study Type	POCUS Intervention	Population	Objective	Sample Size	Results
**Transthoracic Echocardiography**						
Simioniuc et al., 2016 [8]	Retrospective cohort	Doppler echocardiography: mitral inflow velocities	Outpatient HF patients	Outcomes and renal function in outpatients with HFrEF who received echocardiography/BNP-guided therapy vs. standard of care	*n* = 1137	Decreased mortality in the echo–BNP group (HR 0.45, 95%CI: 0.30–0.67, *p* < 0.0001) and decreased rates of renal dysfunction (HR 0.49, 95%CI 0.36–0.67, *p* < 0.0001).
Fletcher et al., 2020 [9]	Systematic review	Tricuspid inflow velocities	Patients with valvular disease, altered RV systolic function, recent cardiac surgery, acute heart failure, heart transplant, pulmonary hypertension, atrial fibrillation	Strength of correlation between E/e’ and RAP	17 articles	Consistent positive correlation between E/e’ and RAP in patients with reduced RV systolic function; equivocal support correlating E/e’ and RAP in heart failure patients.
**Lung US**						
Lichtenstein et al., 2009 [10]	Prospective observational	Lung US	Mechanically ventilated ICU patients	Association between A-line pattern and PCWP	*n* = 102	A-line predominant lung pattern had 90% specificity, 67% sensitivity, 91% positive predictive value, and 65% negative predictive value for diagnosing a PCWP ≤13 mm Hg.
Imanishi et al., 2022 [11]	Prospective cohort	8-zone lung US	Acute HF patients	Association between B-line pattern and PCWP	*n* = 116	1. Clear transition point where PCWP correlated with sudden increase in B-lines: HFrEF, at PCWP = 25 (Δ of 23 B-lines); HFpEF, at PCWP = 19 (Δ of 8 B-lines). 2. Greater than 6 B-lines at discharge resulted in higher risk for cardiac events (HR 12.6, 95% CI: 4.71–33.7; log-rank, *p* < 0.0001).
Coiro et al., 2015 [12]	Prospective cohort	28-zone lung US at discharge	Acute HF patients	Association between B-line pattern and event free survival (all-cause death or HF hospitalization)	*n* = 60	Three-month event-free survival: 27 ± 10% in patients with ≥30 B-lines and 88% ± 5% in those with < 30 B-lines (*p* < 0.0001).
Dubón-Peralta et al., 2022 [13]	Systematic review	Lung US	Acute HF patients	Association between B-line number and hospital readmission/mortality	14 studies	1. More than 30–40 B-lines at admission were a risk factor for readmission or mortality. 2. Persistent B-lines >15 was also a risk factor for readmission or mortality.
Mhanna et al., 2022 [14]	Systematic review and meta-analysis	Lung US-guided diuresis	Outpatient HF patients	HF hospitalization rates and all-cause mortality in HF patients undergoing LUS-guided diuresis vs. standard of care	493 patients across three studies	No significant difference in the rates of heart failure hospitalization between the two groups (RR 0.65; 95% CI 0.34–1.22; *p* = 0.18). No significant difference in all-cause mortality (RR 1.39; 95% CI 0.68–2.82; *p* = 0.37).
Li et al., 2022 [15]	Meta analysis	Lung US-guided diuresis	Outpatient HF patients	Effect of LUS-guided treatment vs. usual care in reducing the major adverse cardiac event (MACE) rate in patients with HF	1203 patients over ten RCT studies	LUS-guided treatment group was associated with a significantly lower risk of MACE compared to standard of care (RR, 0.59; 95% CI: 0.48–0.71; *p* < 0.001).
**Other POCUS**						
CAVA-ADHF (NCT03140566)	RCT	IVC-guided diuresis	Acute HF patients	IVC US measurement compared to clinical assessment to guide diuresis	*n* = 388	Unavailable
JECICA (NCT02892227)	RCT	Cardiac and IVC US	Acute HF patients	Determine if mitral inflow velocities and IVC measurements taken daily will decrease 30-day readmission	*n* = 250	Unavailable
CAVAL US-AHF (NCT04549701)	RCT	IVC and lung US	Acute HF patients	Determine if IVC and LUS decrease subclinical congestion at discharge	*n* = 58	Unavailable
Curbello et al., 2018 [16]	Prospective cohort	IVC collapsibility index	Outpatient HF patients	Association of IVC collapsibility index and worsening HF	*n* = 95	IVCCI <30% and worsening HF: HR of 2.8 (95% CI: 1.3–6.2); IVCCI <30% and hospitalization: HR was 13.9 (95% CI: 1.7–113.0); all-cause mortality—no statistically significant difference.
Pellicori et al., 2019 [17]	Prospective observational	Lung US/IVC US/IJV US and JVD ratio	Outpatient HF patients	Prevalence and clinical significance of subclinical congestion (assessed by ultrasound) in outpatient HF patients	*n* = 203	High levels of N-terminal pro-B-type natriuretic peptide, large IVC diameter, and low JVD ratio were associated with increased hospitalizations/death.
Hossein-Nejad et al., 2016 [18]	Prospective observational	IJV ultrasound: IJV/CCA ratio	Inpatients who underwent central venous catheterization to monitor CVP	Strength of correlation between IJV/CCA and CVP	*n* = 52	1. Significant correlation between IJV/CCA ratio and CVP at inspiration (r = 0.728, *p* < 0.0001) and expiration (r = 0.736, *p* < 0.0001 at expiration). 2. For predicting a CVP <10, IJV/CCA ratio had a sensitivity of 90%, specificity of 86.36%, PPV of 90%, and NPV of 86.36%.
Wang et al., 2022 [19]	Prospective observational	Upright JVP assessment (uJVP)	Adult patients undergoing right heart catheterization for heart failure indications	Strength of correlation between upright assessment of JVP with ultrasound and RAP	*n* = 100	uJVP assessment was 94.6% specific for predicting elevated RAP.
Vaidya et al., 2021 [20]	Prospective cohort	IJV respiratory variation diameter and collapsibility	Heart failure patients undergoing right heart catheterization	Strength of correlation between respiratory variation diameter [(Dmax − Dmin)/Dmax] and IJV collapsibility with RAP	*n* = 72	1. RAP >10 had decreased respiratory variation diameter (14% vs. 40% for IJV, *p* = 0.001) and reduced chance of total collapsibility with sniff (16% vs. 66%, *p* = 0.001). 2. No complete IJV collapsibility had an 84% sensitivity for elevated RAP.
Beaubien-Souligny et al., 2020 [21]	Prospective observational	Hepatic, portal, intra-renal vein Doppler and IVC ultrasound	Cardiac surgery patients	To create a venous excess grading system using ultrasound markers to quantify systemic congestion; association of each grade in the system with AKI	*n* = 145	Significant association between AKI and severe venous congestion grade—defined as IVC >2 and presence of severe flow abnormalities on multiple Doppler patterns (HR: 3.69 CI 1.65–8.24 *p* = 0.001).

2D: two-dimensional; ADHF: acute decompensated heart failure; AKI: acute kidney injury; BNP: B-type natriuretic peptide; CCA: common carotid artery; CI: cardiac index; CO: cardiac output; CVP: central venous pressure; DPAP: diastolic pulmonary artery pressure; DT: E wave deceleration time; HF: heart failure; HFmrEF: heart failure with moderately reduced ejection fraction; HFpEF: heart failure with preserved ejection fraction; HFrEF: heart failure with reduced ejection fraction; HR: hazard ratio; HR: heart rate; IVC: inferior vena cava; JVP: jugular venous pressure; LUS: lung ultrasound; LV: left ventricle; MPAP: mean pulmonary artery pressure; NVP: negative predictive value; PAP: pulmonary artery pressures; PCWP: pulmonary capillary wedge pressure; POCUS: point of care ultrasound; PPV: positive predictive value; PR: pulmonic regurgitation; PVR: pulmonary vascular resistance; RAP: right atrial pressure; RVD: respiratory variation diameter; RVOT: right ventricular outflow tract; SPAP: systolic pulmonary artery pressure; SV: stroke volume; TDI: tissue Doppler imaging; TR: tricuspid regurgitation; TTE: transthoracic echocardiography; VTI: velocity time integral; WU: Wood units.

**Table 2:** Summary of common findings indicative of elevated left ventricular filling pressures across the various POCUS modalities.

Modality	Measure	Normal Range	Interpretation	Limitations
**Cardiac Ultrasound**				
	Mitral E/e’ ratio estimated from the E wave velocity of the mitral inflow Doppler envelope and the tissue Doppler e’ wave velocity at the mitral annulus (septal and/or lateral)	E/e’ ≤ 14	- Values <8 are highly predictive of normal left sided pressures - Elevated E/e’ (>14) is a surrogate for elevated PCWP	- Caution needed in the presence of significant regional wall motion abnormalities, prosthetic mitral valve, annular ring, and significant annular calcification.
	DT in the mitral inflow Doppler envelope	160–200 ms	- DT <40–150 ms suggests elevated PCWP	- Poor estimate of filling pressures in the HFpEF population.
	Peak velocity of the TR jet	≤2/8 m/s	- Elevated velocity suggests increased LV filling pressures	- Signal not always present or clear.
	Estimated PASP using TR jet plus estimated RAP from IVC assessment	≤35 mmHg	- PASP > 45 mmHg suggests increased LV filling pressures	- Caution if extremes in heart rate and for over/under estimation of RAP.
**IVC Ultrasound**				
	Inferior vena cava (IVC) diameter and collapsibility with inspiration	Diameter <21 mm that collapses >50% with sniff	- Normal diameter and response suggest normal RAP (0–5 mmHg) - Diameter > 21 mm with <50% collapsibility suggests elevated RAP (10–20 mm Hg)	- Cannot use in cases of elevated intrabdominal pressures. - May have difficulty obtaining subcostal view due to patient body habitus.
**Lung Ultrasound**				
	Evaluation for B-lines	Absence of B-lines	- In acute settings, >30 lines (in 28-zone scanning field) indicate lung congestion - Persistent presence of B-lines indicates residual congestion	- Mostly used and validated in acute settings; less useful in outpatient management of heart failure.
**IJV Ultrasound**				
	Ratio of the maximum cross-sectional diameter of the IJV during Valsalva to the diameter at rest (JVD ratio)	JVD ratio ≥4	- Ratio < 4 is abnormal and <2 may indicate severe congestion	- Requires a high-frequency linear (vascular) transducer.

DT: E wave deceleration time; HFpEF: heart failure with preserved ejection fraction; IJV: internal jugular vein; IVC: inferior vena cava; JVD: internal jugular vein diameter; LV: left ventricle; PASP: pulmonary artery systolic pressure; PCWP: pulmonary capillary wedge pressure; POCUS: point of care ultrasound; RAP: right atrial pressure; TR: tricuspid regurgitation.

## Data Availability

Not applicable
